# Practical NIR Assay Derived from Cyanine to Evaluate Intracellular H_2_S in Living Cell Imaging

**DOI:** 10.3390/s24123744

**Published:** 2024-06-08

**Authors:** Chenqian Ye, Axue Wang, Yuxin Lu, Xinye Lin, Luqiang Huang, Daliang Li

**Affiliations:** 1 College of Life Sciences, Fujian Normal University, Fuzhou 350117, China; qsx20211390@student.fjnu.edu.cn (C.Y.); qsz20231751@student.fjnu.edu.cn (A.W.); qsx20231416@student.fjnu.edu.cn (Y.L.); lxy1978377397@163.com (X.L.); 2 Fujian Key Laboratory of Innate Immune Biology, Biomedical Research Center of South China, Fujian Normal University, Fuzhou 350117, China

**Keywords:** fluorescent probe, hydrogen sulfide, near infrared

## Abstract

To monitor the biological function of H_2_S in real time, this investigation demonstrated the design and synthesis of a novel fluorescent probe integrated with cyanine and 2,4-dinitrophenol for the qualitative and quantitative detection of H_2_S. An NIR sensitive sensor (FS-HS-1) was provided with a straightforward process. Spectroscopy experiments elucidated that FS-HS-1 could selectively detect H_2_S in a PBS solution (containing 40% acetonitrile) with a 111-fold fluorescence enhancement at 715 nm (ex. 605 nm). The response towards NaHS occurred in less than 2 min, and the detection limit was confirmed to be as low as 4.47 ± 0.11 nmol/L. Furthermore, the probe is capable of monitoring changes in exogenous H_2_S concentrations within living cells with confocal and 2P imaging.

## 1. Introduction

Hydrogen sulfide (H_2_S) holds a notable status as an endogenous, physiological regulator, playing crucial roles in mammalian cells and tissues [1,2]. Enzymes like cystathionine β-synthase (CBS), cystathionine γ-cleaving enzyme (CSE), and 3-mercaptopyruvate sulfurtransferase (3-MST) are responsible for its production in mammalian systems. H_2_S is involved in a myriad of physiological processes, such as vascular smooth muscle relaxation [3,4], neurotransmission, insulin signaling [5], inflammation regulation [6], regulation of gene transcription and translation, cellular bioenergetics [7], metabolism, immune function, and the regulation of various functions of the central and peripheral nervous systems, among others, making it pertinent for physiologists and clinicians [8]. Furthermore, its implications extend to the realms of cancers [9] and neurological diseases [10], positioning hydrogen sulfide as a significant gas mediator alongside nitric oxide and carbon monoxide gases [11,12]. Techniques enabling the monitoring of regulated secretion dynamics with high sensitivity and temporal precision are indispensable for advancing such investigations.

Various detection methods for hydrogen sulfide are currently employed, encompassing visual analysis, fluorescence analysis, and electrochemical analysis [13]. Among them, fluorescence-based techniques have garnered widespread attention due to their simplicity, high sensitivity, low cost, and real-time detection capabilities. The increasing demand for specialized and sensitive fluorescent probes with rapid response to hydrogen sulfide under gentle conditions underscores their critical role in enhancing our comprehension of H_2_S’s biological functions [14,15,16,17]. Despite their significance, practical limitations persist in the application of probes, such as prolonged response times and inadequate emission excitation wavelengths. Thus, proactive development of probes with practical potential remains paramount. Recent endeavors have shown promise by introducing novel near-infrared fluorophores and detailing their applications [18,19,20,21].

The detection mechanism of most H_2_S sensors hinges on the potent quenching effect of nitro, which eliminates the electron-withdrawing 2,4-dinitrophenyl (DNP) group from the non-fluorescent probe in the presence of H_2_S, eliciting hyperfluorescence. Typically, dinitrophenyl (DNP) ether thiolysis can be readily incorporated into the probe’s structure through a single-step reaction [17]. Notably, the thiolysis of H_2_S by this recognition group surpasses that of biothiols like GSH and Cys, ensuring heightened probe selectivity for H_2_S. Although many H_2_S fluorescent probes based on DNP sulfurization have been published and have excellent performance, a lot of effort is still necessary to better signal and accelerate reaction time. Consequently, in this study, we opted to employ 2,4-dinitrophenol as the probe’s recognition group in the strategy.

Drawing inspiration from the QCy-7 strategy outlined in the prior literature [22,23], which involves a structural shift in NIR probes utilizing a masking group like 2,4-dinitrobenzene, we set out to innovate novel fluorescent probes following a similar avenue. Capitalizing on the favorable quantum yield and near-infrared emission wavelength of QCy-7 dye, 2,4-dinitrofluorobenzene was chosen as the recognition group to synthesize a fresh fluorescent probe, FS-HS-1, customized for H_2_S detection. Given that H_2_S exists primarily in its HS^-^ form at physiological pH [24], this investigation employed NaHS as the H_2_S donor, analyzing structural alterations of FS-HS-1 pre- and post-addition of NaHS to provoke distinct fluorescence characteristics. Through this exploration, we successfully identified a near-infrared probe, FS-HS-1, proficient in detecting H_2_S with remarkable sensitivity (detection limit of 4.47 ± 0.11 nmol/L), fast response (<2 min), high specificity (unaffected by sulfur dioxide derivatives), and a remarkable 111-fold fluorescence intensity surge. Noteworthy cellular experiments further validated the effectiveness of probe FS-HS-1 in H_2_S imaging within cells with confocal and 2P imaging. The discernible changes in fluorescence intensity act as a distinctive signal for intramolecular H_2_S detection, facilitating successful cellular imaging of exogenous H_2_S by FS-HS-1. Compared with the reported probes masked with DNP, most of them suffered from intricate synthesis processes, or visible region emission, where their fluorescence imaging would be susceptible to interference from cellular autofluorescence (<700 nm) [25,26,27,28,29,30,31] or long response times [19,32]. The endeavor based on FS-HS-1 might contribute to constructing the complementary assay with satisfactory properties after the further optimization.

## 2. Materials and Methods

### 2.1. Materials

4-Hydroxyisophthalaldehyde (Bide Pharmaceutical Technology Co., Ltd., Shanghai, China); 2,4-dinitrofluorobenzene (West Asia Reagent Co., Ltd., Shandong, China); lysine (Sinopharm Chemical Reagent Co., Ltd., Shanghai, China); tyrosine (Sinopharm Chemical Reagent Co., Ltd., Shanghai, China); methionine (Sinopharm Chemical Reagent Co., Ltd., Shanghai, China); sodium sulfite (Sinopharm Chemical Reagent Co., Ltd., Shanghai, China); sodium bi sulfite (Sinopharm Chemical Reagent Co., Ltd., Shanghai, China); sodium sulfate (Sinopharm Chemical Reagent Co., Ltd., Shanghai, China); sodium sulfate (Sinopharm Chemical Reagent Co., Ltd., Shanghai, China); sodium dithionite (Sinopharm Chemical Reagent Co., Ltd., Shanghai, China); sodium metabisulfite (Sinopharm Chemical Reagent Co., Ltd., Shanghai, China); 30% hydrogen peroxide solution (Xi long Chemical Co., Ltd., Guangxi, China); sodium hypochlorite solution (Xi long Chemical Co., Ltd., Guangxi, China); sodium acetate (Guangdong Guanghua Science and Technology Co., Ltd., Guangdong, China); sodium nitroprusside (Shanghai Aladdin Biochemical Technology Co., Ltd., Shanghai, China); N-ethylmaleimide (Thermo Fisher Scientific, Waltham, MA, USA).

Rotary Evaporator (EYELA Corporation, Tokyo, Japan); Nuclear Magnetic Resonance Spectrometer (Bruker, Madison, WI, USA); LCQ-FLEET Mass Spectrometer, (Thermo Fisher Scientific, Waltham, MA, USA); UV-1900 Dual-Beam UV–Vis Spectrophotometer (Shimadzu, Tokyo, Japan); FS5 Fluorescence Spectroscopy (Edinburgh Instruments, Edinburgh, England); Multifunctional Enzyme Labeling Instrument (BMG FLUOstar OPTIMA, Offenburg, Germany); Nitrogen dioxide incubator (Thermo Fisher Scientific, Waltham, MA, USA); Laser confocal microscope (ZEISS, Oberkochen, Germany).

### 2.2. Synthesis of Probe FS-HS-1

1,1,2-Trimethyl-1H-benzo [e]indole (10 mmol, 2.09 g) was dissolved in a 5 mL solution of acetonitrile in a 100 mL round-bottomed flask, followed by the gradual addition of iodomethane solution (10 mmol, 0.625 mL). The flask was then positioned in an oil bath at 80 °C and refluxed for 2 h. After completion of the reaction, the solution was cooled to room temperature and poured into a petroleum ether solution, undergoing repeated washes until the solution turned colorless and transparent. The resulting pellet was collected, filtered, and dried to obtain 1.456 g of light gray powder compound **1a** (The yield was 64%), which was considered pure enough for subsequent reaction steps.

In another 100 mL round-bottom flask, 4-hydroxyisophthalaldehyde (300 mg, 2 mmol), 2,4-dinitrofluorobenzene (372 mg, 2 mmol), and K_2_CO_3_ (414 mg, 3 mmol) were combined in a 5 mL solvent of DMF and allowed to react at room temperature for 1 h. Subsequently, 50 mL of ethyl acetate was added for dilution, followed by washing with water (30 mL × 3) [33]. The solution was then dried using sodium sulfate and subjected to spin drying to eliminate the solvent, resulting in 293.26 mg of compound **1b** (The yield was 46.3%), meeting purity requirements for the upcoming reaction step.

**Scheme 1:**
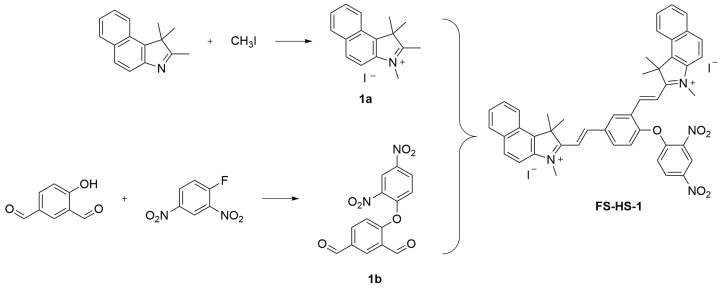
Synthesis route of probe FS-HS-1.

For the final step, compound **1a** (48 mg, 0.15 mmol), compound **1b** (105 mg, 0.3 mmol), and sodium acetate (37 mg, 0.45 mmol) were mixed in 2.5 mL of acetic anhydride solution and refluxed at 80 °C under argon for 2 h. The reaction mixture was then evaporated under reduced pressure, followed by separation and purification through silica gel column chromatography (dichloromethane/methanol = 10:1). The product, probe FS-HS-1, was obtained in a yield of 27.52%, yielding 30 mg of the final product (Scheme 1). ^1^H NMR (600 MHz, DMSO-*d*_6_) δ 9.18 (s, 1H), 9.01 (s, 1H), 8.59 (d, *J* = 15.8 Hz, 3H), 8.47–8.38 (m, 3H), 8.32 (s, 2H), 8.22 (s, 2H), 8.15 (s, 2H), 7.99 (d, *J* = 16.7 Hz, 1H), 7.89 (d, *J* = 16.6 Hz, 1H), 7.80 (d, *J* = 22.9 Hz, 2H). HRMS (high resolution mass spectra) was performed: 727.2915 calculated for C_46_H_39_N_4_O_5_^+^ (M-2I-H)^+^; observed: 727.2921 (M-2I-H)^+^; and 364.1494 calculated for C_46_H_40_N_4_O_5_^2+^ (M-2I)^2+^; observed: 364.1496 (M-2I)^2+^ (Figures S1–S3).

The synthesis of the probe FS-HS-2 was described in the supporting information (Scheme S1). The construction and characterization of FS-HS-1-P was performed in in the supporting information (Scheme S2, Figures S5–S7).

### 2.3. Selective Analysis Experiments

An amount of 3 mL of aqueous solution (containing 40% acetonitrile in PBS), along with 3 μL of probe solution (10 mM in DMSO), and 6 μL of different interferents (100 mM in PBS) were added to the cuvette and mixed thoroughly. Subsequently, the UV absorption and fluorescence spectra were detected by a UV-Vis spectrophotometer and fluorescence spectrophotometer, respectively. The excitation wavelength was 605 nm, and the slit width was 4 nm.

### 2.4. Reaction Time Analysis Experiments

The mixture of 3 mL of an aqueous solution (containing 40% acetonitrile in PBS), the probe solution and NaHS solution was integrated together and mixed thoroughly. The fluorescence intensity at 715 nm was recorded once every 2 min, with the same parameters as in Section 2.3, to obtain the fluorescence spectratime relationship graph.

### 2.5. Titration Experiment

The mixture of 3 mL of an aqueous solution (containing 40% acetonitrile in PBS), 3 μL of the probe solution, and NaHS solution was integrated and thoroughly mixed together. The fluorescence spectra were recorded, using the same parameters as in Section 2.3, to obtain titration curve.

### 2.6. Potential Applications in Cells

Cytotoxicity: Adherent cells with robust growth were carefully selected, and the old medium was removed. Following trypsin digestion, the cells were resuspended in 2 mL of fresh medium, aspirated, and then centrifuged. Post-centrifugation, the cells were quantified to determine a cell density of 8000 cells per well. The cells were then plated into 96-well plates and incubated at 37 °C for 24 h in a CO_2_ incubator. After discarding the old medium, 100 μL of fresh medium containing varying concentrations of probes was added to each well, with 6 replicates per concentration group. Additionally, a blank control group (medium only) and a control group (medium with cells) were included for comparison, followed by continuing the incubation process.

Upon completion of the incubation period, the old medium was aspirated, and fresh medium along with CCK-8 reagent was added to each well. The incubation continued for an additional 3 h. Subsequently, the absorbance at 450 nm was recorded using an enzyme marker, enabling the calculation of the corresponding cell viability.

Confocal imaging (single-photon experiments): Cells were inoculated in fluorescent dishes and cultured in confocal dishes at a cell density of 1.2–1.5 w/dish for 24 h. The old medium was aspirated and washed by adding HBSS solution, followed by incubation with the probe (10 μM) for 30 min for imaging. After the probe incubation was finished, different concentrations of NaHS were added to react for 30 min. The excitation wavelength was 543 nm, and the emission wavelength was 700–750 nm.

Two-photon imaging: (a) Exogenous experiments: Firstly, a 6 cm cell culture dish was selected, and the cell culture medium was removed. Add 2 mL of medium containing the probe (10 μM) and incubate for 30 min away from light. After incubation, wash the cell culture dish twice with DPBS solution. Finally, 7 mL of Hank’s solution was added to the cell culture dish, and then NaHS with different concentration gradients was added to the cell culture dish for confocal photography. (b) Endogenous experiments: according to the method in the literature [34], the experimental operation was performed by first adding SNP and probe incubation for 30 min, followed by probe incubation for 40 min, and then adding NaHS solution incubation for 30 min for confocal imaging. The excitation wavelength was 800 nm, and the emission wavelength was 700–750 nm.

## 3. Results and Discussion

### 3.1. Selective Analysis Experiments

**Figure 1:**
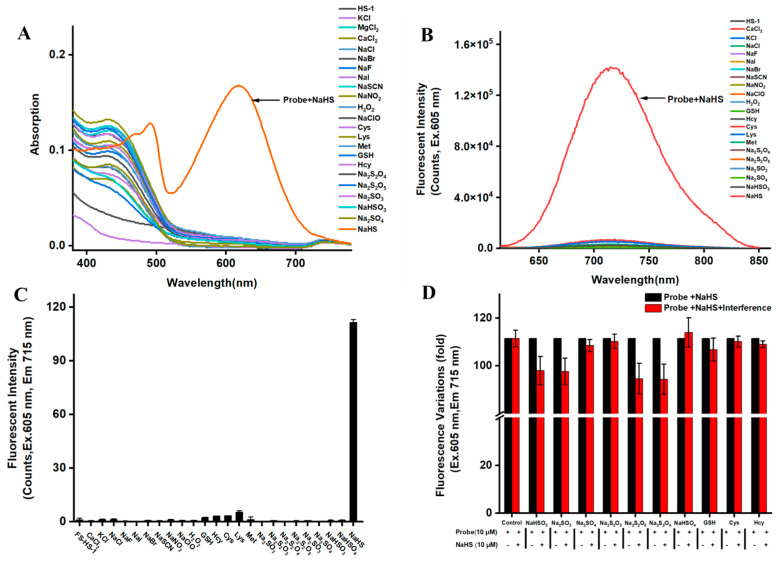
Behavior of FS-HS-1 (10 μM) with different analytes (Ca^2+^, K^+^, Na^+^, F^−^, Br^−^, I^−^, SCN^−^, NO_2_^−^, ClO^−^, H_2_O_2_, GSH, Hcy, Cys, Lys, Met, SO_3_^2−^, SO_4_^2−^, S_2_O_4_^2−^, S_2_O_5_^2−^, HSO_3_^−^) at 200 μM. Fluorescence spectrum of FS-HS-1 with interferents (**A**); absorption spectrum (**B**); fluorescence histograms of selectivity (**C**); interference experiments between the probe (10 μM), NaHS (20 μM) and different sulfur species (100 μM), “+” indicates that it was added, and “-” indicates that it was not added (*n* = 3) (**D**).

The capability of the fluorescent probe to maintain specific analyte detection in practical scenarios was a crucial factor for evaluating its effectiveness. Therefore, we investigated the probe’s response to potential interfering substances. By examining the fluorescence signal of the 10 μM probe when exposed to various analytes (Ca^2+^, K^+^, Na^+^, F^−^, Br^−^, I^−^, SCN^−^, NO_2_^−^, ClO^−^, H_2_O_2_, GSH, Hcy, Cys, Lys, Met, Tyr, SO_3_^2−^, SO_4_^2−^, S_2_O_3_^2−^, S_2_O_4_^2−^, S_2_O_5_^2−^, HSO_3_^−^, HSO_4_^−^), we aimed to assess its selectivity. The emergence of a new absorption peak at 605 nm and an emission peak at 715 nm only occurred in the presence of NaHS. A histogram was constructed using 605 nm excitation light, the fluorescence enhancement at 715 nm as the vertical axis, and different interferents as the horizontal axis (Figure 1A,B). The results, as depicted in Figure 1C, indicated that the probe exhibited minimal reactivity towards the interferents tested, whereas the fluorescence enhancement with NaHS at a concentration of 20 μM reached a remarkable 111-fold increase. UV and fluorescence spectroscopy analyses revealed consistent fluorescence intensity before and after exposure to the interferents, affirming the high selectivity of the probe FS-HS-1. It could be observed that the fluorescence of FS-HS-1 (10 μM) at 715 nm increased approximately 111-fold after the probe reacted with NaHS (20 μM) (Figure 1C). It has been reported in the literature that the removal of dinitrophenol groups under alkaline conditions was performed with thiol as a thiolytic solvent [35]. The pKa of H_2_S was 6.9, and the pKa of glutathione and cysteine was about 8.5. Therefore, under physiological conditions, the probe’s selectivity for H_2_S will be higher than that of biological mercaptans like GSH and Cys. When other sulfur species were added, the fluorescence intensity decreased slightly, but the overall interference was not significant. FS-HS-2 did not show a significant response to different biological thiols (Hcy, Cys, GSH), and it also lacked selectivity and sufficient detection capacity for NaHS (Figure S8B). These results indicate that FS-HS-1 has high specificity towards NaHS (Figure 1D).

### 3.2. Response Time and pH Experiments

**Figure 2:**
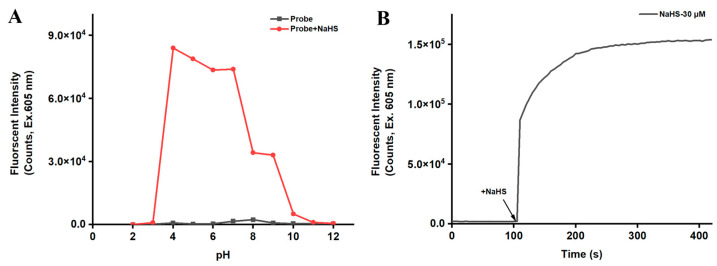
Comparison of FS-HS-1 (10 μM) with and without NaHS in various pH conditions and the temporal kinetics. The tendency of probe FS-HS-1 with different pH conditions (**A**); temporal kinetics of FS-HS-1 towards NaHS (30 μM) (**B**).

Shown in Figure 2A, the optimal pH of the probe reacted with NaHS might range from 4 to physiological pH conditions because the fluorescence intensity of FS-HS-1 towards NaSH maintained relatively stable in this range. With PBS (40% acetonitrile) solution as the reaction agent, the fluorescence intensity of the probe itself had almost no significant change under different pH conditions. The results disclosed that the resulted product might have a wide range of applications as a pH-center and could be used for environmental and biological detection.

Analyzing Figure 2B revealed that the initial fluorescence intensity of the probe at 715 nm in an aqueous solution (PBS, pH 7.4, with 40% acetonitrile) was relatively low. However, under 605 nm excitation, the fluorescence intensity at 715 nm notably increased following reactions with varying concentrations of NaHS. The experimental results clearly indicated the probe’s prompt response to NaHS, achieving complete detection within 2 min. In contrast, probe FS-HS-2 exhibited a 30 min response time to NaHS (Figure S8A). Based on this comparison, probe FS-HS-1 was selected for subsequent investigations.

### 3.3. Titration Experiments of FS-HS-1

**Figure 3:**
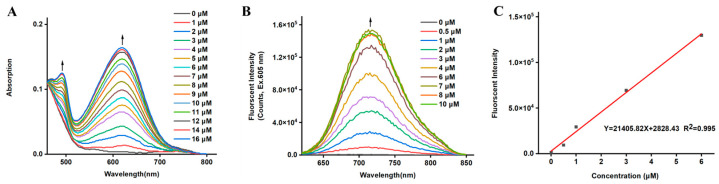
Titration curve of FS-HS-1 towards NaHS. Absorption spectrum (**A**), fluorescence spectrum (**B**), and linear relationship (**C**) (*n* = 3); The arrows indicate an increase or decrease in fluorescence intensity or UV absorption at the corresponding wavelength.

In our subsequent investigation, we delved into the fluorescence response of FS-HS-1 to varying concentrations of NaHS to understand its concentration-dependent characteristics. As the NaHS concentration rose, the absorption value of the probe at 610 nm steadily increased (Figure 3A). The fluorescence intensity of the probe at 605 nm exhibited a linear response to the HS- concentration within the range of 0–6 μM (Figure 3B), demonstrating a strong linear relationship with a correlation coefficient of 0.995 (Figure 3C). The calculated limit of detection (LOD) stands at 4.47 ± 0.11 nmol/L. These results revealed that FS-HS-1 displays heightened sensitivity to NaHS concentration, offering promising applications in various fields.

### 3.4. Proposed Mechanism of FS-HS-1 towards H_2_S

**Scheme 2:**
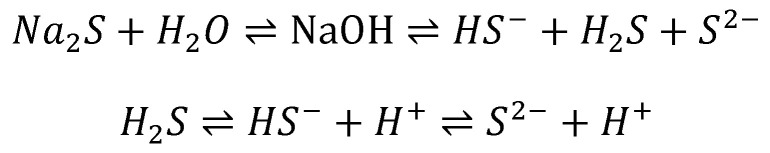
Forms of NaHS and Na_2_S in water.

Given the efficient detection capabilities of FS-HS-1 for NaHS, we proceeded to employ it as a subsequent probe for further examination. Our next step involved investigating the reaction mechanism of probe FS-HS-1. Typically, H_2_S donors comprise water-soluble sulfide salts like NaHS and Na_2_S. When these sulfides were dissolved in aqueous solutions, they rapidly decomposed under physiological pH conditions to generate HS^−^ and H_2_S, establishing a dynamic equilibrium within living organisms. Primarily existing in the anionic form HS^−^, these divalent sulfur elements possessed robust reducing properties (Scheme 2).

**Scheme 3:**
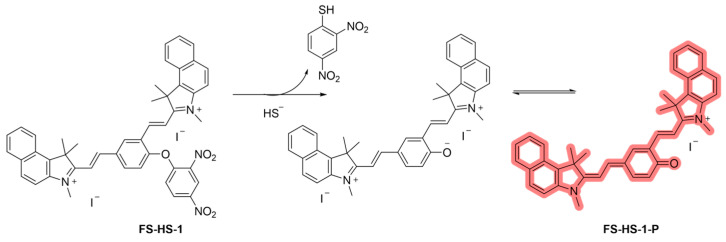
The sensing mechanism of NaHS sensing by FS-HS-1.

As per both literature reference [36] and experimental findings, it was established that 2,4-dinitrobenzene served as a fluorescence-quenching electron acceptor, obstructing the intramolecular photoinduced electron transfer process upon binding to fluorescent dyes. Subsequently, the probe adopted a cage conjugation mode, rendering the probe itself non-fluorescent [37]. Upon nucleophilic interaction with the thiol group, the probe released Cy-phenolate and thiol-dinitrobenzene. The Cy-phenolate underwent rearrangement by neutralizing the positive charge on the nitrogen atom to form a stable Cy-quinone. This Cy-quinone reinstated the extended π-electron conjugation mode of the elanine dyes, thereby activating NIR fluorescence. Drawing upon the prior literature and experimental observations, a hypothesized response mechanism of probe FS-HS-1 to H_2_S was proposed (Scheme 3).

**Figure 4:**
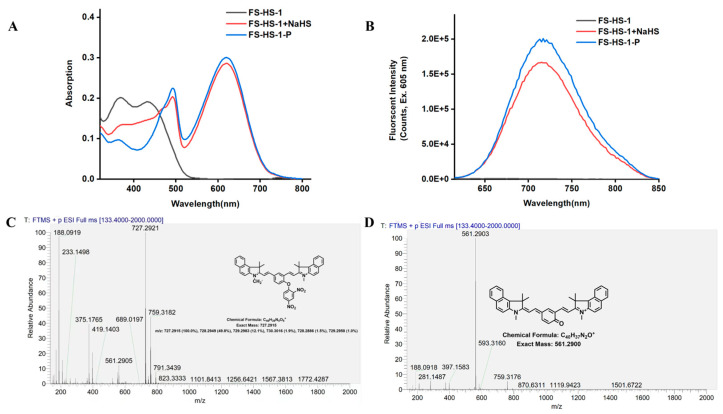
Photophysical properties and HRMS of FS-HS-1, FS-HS-1 with NaHS and FS-HS-1-P. Ultraviolet (**A**) and fluorescence spectra (**B**) of FS-HS-1-P before and after reaction with NaHS and FS-HS-1-P; HRMS: probe FS-HS-1 (**C**); Probe FS-HS-1 with NaHS (**D**).

The spectral results of Figure 4A,B showed that the absorption and emission peaks of the probe FS-HS-1 after reacting with NaHS were consistent with those of FS-HS-1-P, which also confirms our speculation on the probe detection mechanism. The results from mass spectrometry (high resolution mass spectra, HRMS) analysis in Figure 4C,D showed that the resulted main peak at *m*/*z* = 561.3019 (C_40_H_37_N_2_O^+^) of FS-HS-1 with NaHS could be aligned with the predicted cationic pattern, denoted as FS-HS-1-P. Meanwhile, probe FS-HS-1 could be observed as a promising value at *m*/*z* = 727.2921 (C_46_H_39_N_4_O_5_^+^, [M-2I-H] ^+^) before the reaction with NaHS. All the information was consistent with the proposed mechanism according to the literature references [36,37].

### 3.5. Potential Application of Probe FS-HS-1 in Cells

#### 3.5.1. Cytotoxicity Assay

**Figure 5:**
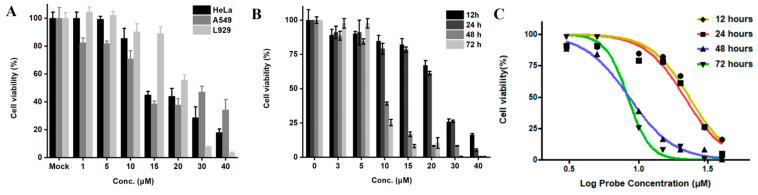
Cytotoxicity results of the probe FS-HS-1. A comparison of cytotoxicity assays (**A**); the toxicity monitoring of L929 cells from 0 to 72 h (**B**); and the calculations of IC_50_ (**C**).

To explore the cytotoxicity of the probe, FS-HS-1 were incubated in HeLa cells, A549 cells, and L929 cells for 6 h (Figure 5A). Among them, it was obvious that L929 cells should exhibit better tolerance to the probe. Thus, L929 cells were considered as the object of continuous incubation and imaging of subsequent probes to understand the potential of FS-HS-1.

In the further investigations about biocompatibility, the impact of FS-HS-1 on the viability of L929 cells was concerned with the prolonged incubations over 1–3 days. As shown in Figure 5B, cell viability gradually decreased with increasing incubation time and probe concentration. Notably, the 10 μM probe-maintained cell viability above 80% after 24 h (hours) of incubation, meeting the concentration requirement for cell imaging experiments. The IC_50_ values at different time points were determined to be 23.25 μM, 21.52 μM, 8.55 μM, and 8.36 μM, respectively, based on the relationship between incubation times and probe-induced cell lethality. These results supposed that under the presented imaging conditions, particularly at a concentration of 10 μM, FS-HS-1 exhibited promising biocompatibility when the incubation time was less than 24 h (Figure 5C).

#### 3.5.2. Single-Photon Cell Imaging Experiment

**Figure 6:**
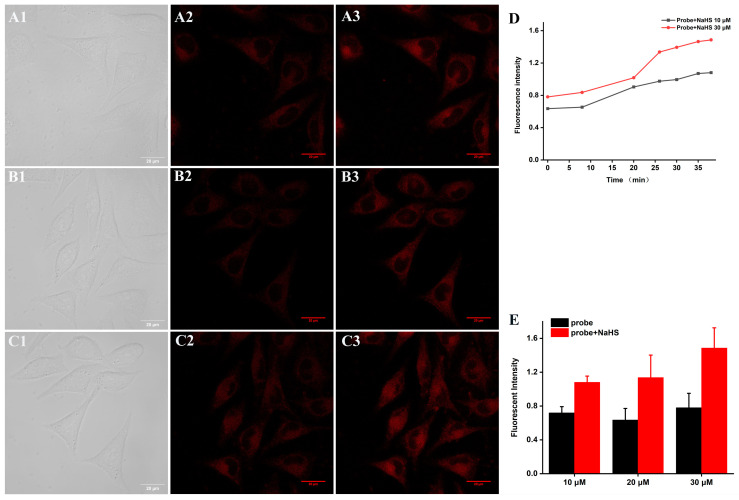
Confocal imaging of L929 cells with probe FS-HS-1 (10 μM) at different concentrations of NaHS (10 μM, 20 μM, 30 μM). Red channel: Ex. 543 ± 20 nm, Em. 700 ± 20 nm, scale bar = 20 μm. After staining with FS-HS-1, cell treated with 10 μM (**A**), 20 μM (**B**) and 30 μM (**C**) of NaHS (1: Bright field; 2: Fluorescence image after 30 min of probe FS-HS-1 staining; 3: Fluorescence image after staining with the addition of hydrogen sulfide for 30 min); Fluorescence intensity changes in FS-HS-1 and L929 cells at different concentrations of NaHS over 0–38 min (**D**); Fluorescence enhancement folds of FS-HS-1 and L929 cells at different concentrations of NaHS (10–30 μM) (**E**).

Subsequently, an assessment was conducted to determine the capability of FS-HS-1 (10 μM) to facilitate fluorescence imaging of NaHS within living cells. L929 cells were incubated with FS-HS-1 for 30 min, followed by treatment with varying concentrations of NaHS solution for an additional 30 min (Figure 6A,B). The staining results from these cell samples demonstrated the probe’s ability to detect exogenous NaHS within living cells and to track changes in NaHS levels through fluorescence alterations.

Observations from Figure 6D,E revealed that the fluorescence intensity of the probe, upon interacting with different NaHS concentrations, stabilizes around 35 min. Specifically, at concentrations of 10 μM and 30 μM, the fluorescence intensity increased by approximately 1.12-fold and 1.53-fold, respectively. Evidently, the fluorescence intensity of the probe exhibited a gradual ascent in response to escalating concentrations of external NaHS solution.

#### 3.5.3. Two-Photon Cell Imaging Experiment

**Figure 7:**
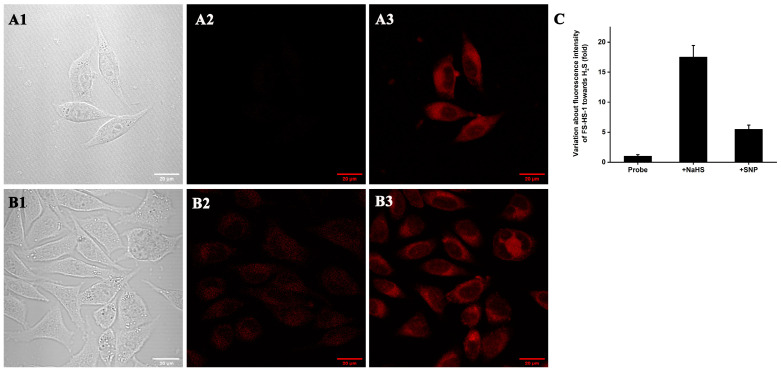
2P Confocal imaging of L929 cells with probe FS-HS-1 (10 μM). Red channel: λ_ex_ = 800 nm, λ_em_ = 700–750 nm, scale bar = 20 μM. After staining with FS-HS-1, cell treated with NaHS (**A**), SNP (**B**) (1: Bright field; 2: Fluorescence image after 30 min of probe FS-HS-1 staining; 3: After 30 min incubation with probe FS-HS-1, fluorescence image after 30 min incubation of cells with the additives); Fluorescence variations of FS-HS-1 towards exogenous and endogenous H_2_S (**C**).

From the results presented in Figure 7, it was evident that upon incubation of the probe with L929 cells, a faint fluorescence signal was observed within the cells. Subsequent addition of NaHS to the L929 cells resulted in a significant increase in fluorescence intensity, with a notable enhancement reaching approximately 17 times. The following investigation focused on the probe’s experimental impact on detecting endogenous H_2_S and its inhibitory properties. Sodium nitroprusside (SNP) was used to trigger H_2_S production in cells, allowing the reaction with the probe to test its ability to detect the generated H_2_S [38]. Upon incubating L929 cells with the probe for 30 min, a weak fluorescent signal was observed within the cells. The fluorescence intensity increased by 5.5 times following a 30 min incubation with the addition of nitroprusside (SNP). Compared with the other similar sensors equipped with DNP, FS-HS-1 exhibited excellent sensitivity (LOD at 4.47 ± 0.11 nmol/L), satisfactory response time, and rational 2P imaging potentials (Table S1).

## 4. Conclusions

This investigation introduced a novel turn-on fluorescent probe, FS-HS-1, designed for the selective detection of H_2_S. Formulated with the structural elements of 2,4-dinitrobenzene and cyanine dyes, FS-HS-1 offered advantages such as a low detection limit, rapid reaction time, and high selectivity. With a detection limit for H_2_S set at 4.47 ± 0.11 nmol/L, the probe showcased remarkable sensitivity towards H_2_S detection. FS-HS-1 had been effectively utilized for fluorescence imaging of exogenous hydrogen sulfide within cellular environments. This paper unveiled a groundbreaking fluorescent probe tailored for the identification of hydrogen sulfide signaling molecules in cells, enabling the exploration of endogenous H_2_S signaling within cellular contexts and opening avenues for future in vivo imaging studies.

## Data Availability

Data are available on request from the corresponding author.

## Supplementary Materials

The following supporting information can be downloaded at https://www.mdpi.com/article/10.3390/s24123744/s1: Figure S1: ^1^HNMR of FS-HS-1; Figure S2: ^13^CNMR of FS-HS-1; Figure S3: HRMS of FS-HS-1; Figure S4: ^1^HNMR of FS-HS-2; Figure S5: ^1^HNMR of FS-HS-1-P; Figure S6: ^13^CNMR of FS-HS-1-P; Figure S7: HRMS of FS-HS-1-P; Figure S8: Behaviors of FS-HS-2 (10 μM) towards NaHS (100 μM): The response times (**A**); Fluorescence images of FS-HS-2 on biothiols (100 μM), respectively (**B**); Figure S9: Colorimetric behaviors of FS-HS-1 (10 μM) towards NaHS (0, 5, 10, 15, 25, 50, 100 μM); Table S1: Comparison of FS-HS-1 with the reported H_2_S-specific fluorescent probes; Scheme S1: Synthesis route of probe FS-HS-1; Scheme S2: Forms of NaHS and Na_2_S in water.
