# Supplementary material for: Practical NIR Assay Derived from Cyanine to Evaluate Intracellular H2S in Living Cell Imaging
Source: Sensors (Basel). 2024 Jun 8;24(12):3744. doi: 10.3390/s24123744 (PMC11207956; doi:10.3390/s24123744)
Supplement: Supplementary file 1 [file sensors-24-03744-s001.zip › sensors-2978398-supplementary.pdf]

Practical NIR Assay Derived from Cyanine to Evaluate Intracellular H_2S in Living Cell Imaging

Chenqian Ye [1, 2], Axue Wang [1, 2], Yuxin Lu [1, 2], Xinye Lin [1], Luqiang Huang [1*], Daliang Li [1, 2*]

[1]College of Life Sciences, Fujian Normal University, Fuzhou, China;
[2]Fujian Key Laboratory of Innate Immune Biology, Biomedical Research Center of South China, Fujian Normal University, Fuzhou, China;
*Correspondence: biohlq@ fjnu.edu.cn; daliangli@fjnu.edu.cn

1. Characterization of FS-HS-1
2. Synthesis and characterization of FS-HS-2
3. Synthesis and characterization of FS-HS-1-P
4. Spectroscopic experimental results of FS-HS-2
5. Colorimetric potential of FS-HS-1
6. Comparison with the reported H2S-specific fluorescent probes

1. Characterization of FS-HS-1

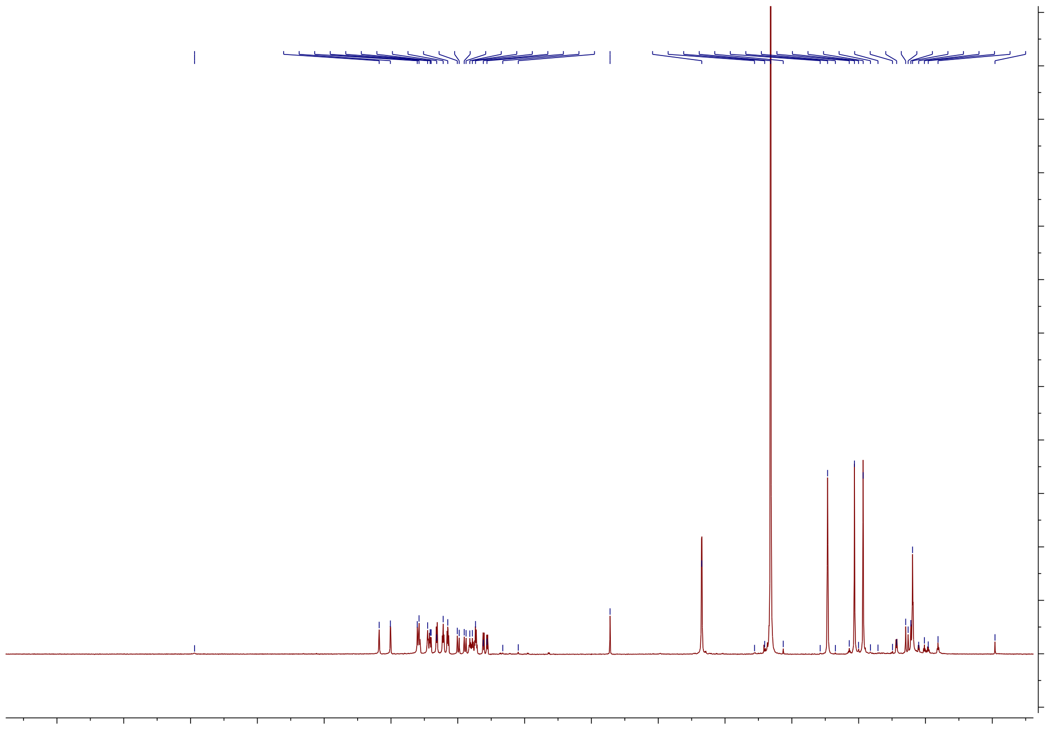

Figure S1 ¹HNMR of FS-HS-1

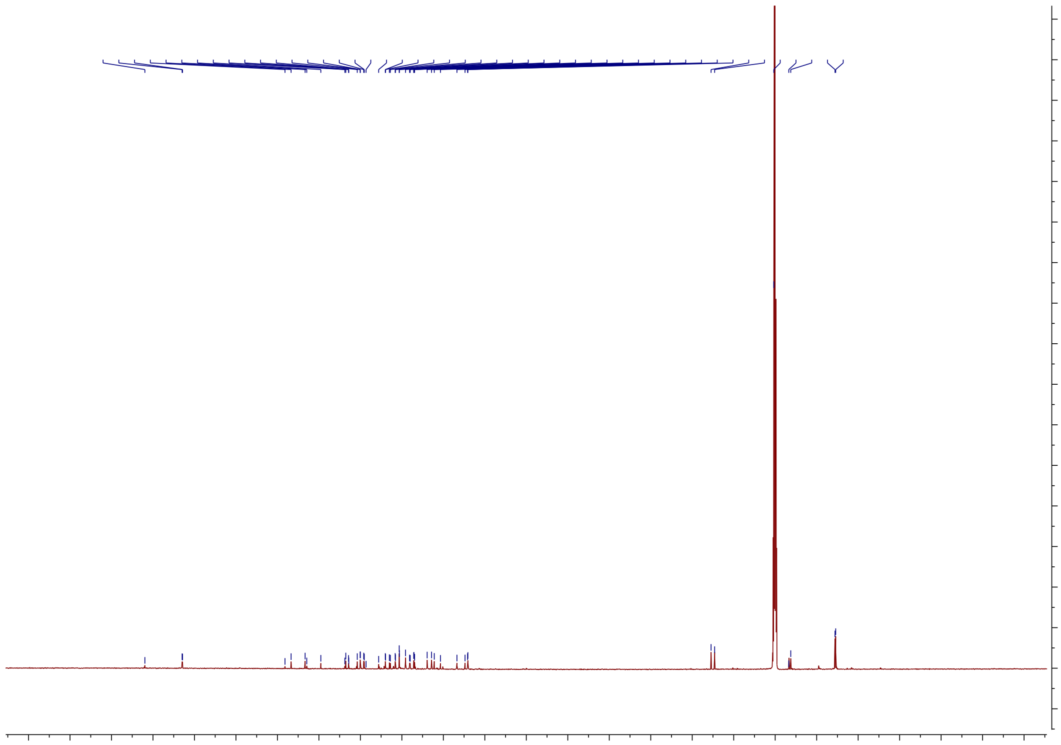

Figure S2 ¹³CNMR of FS-HS-1

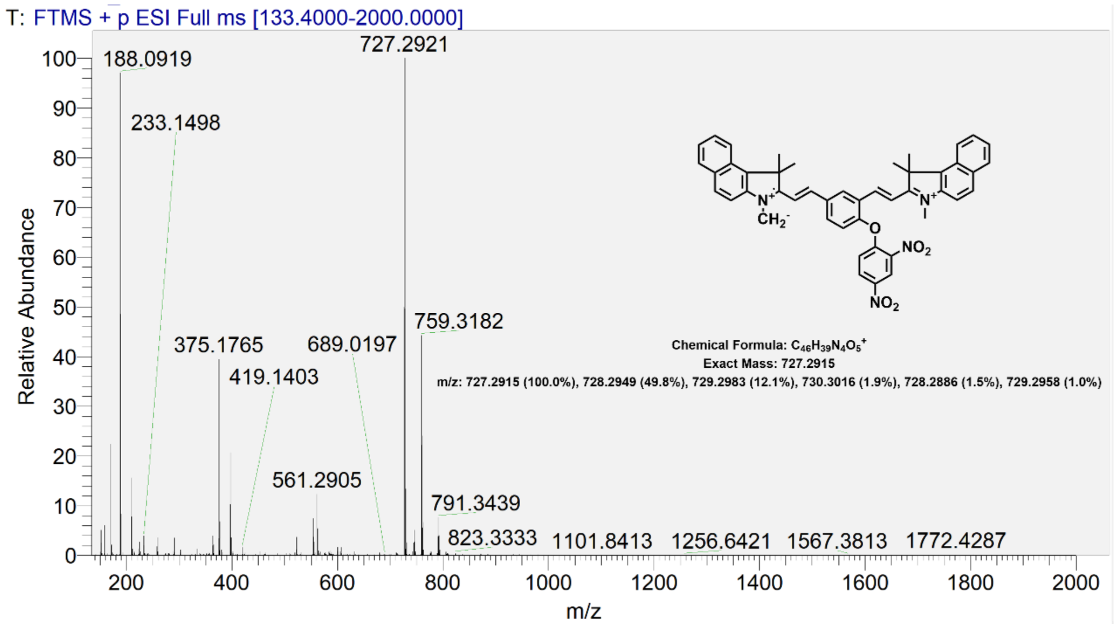

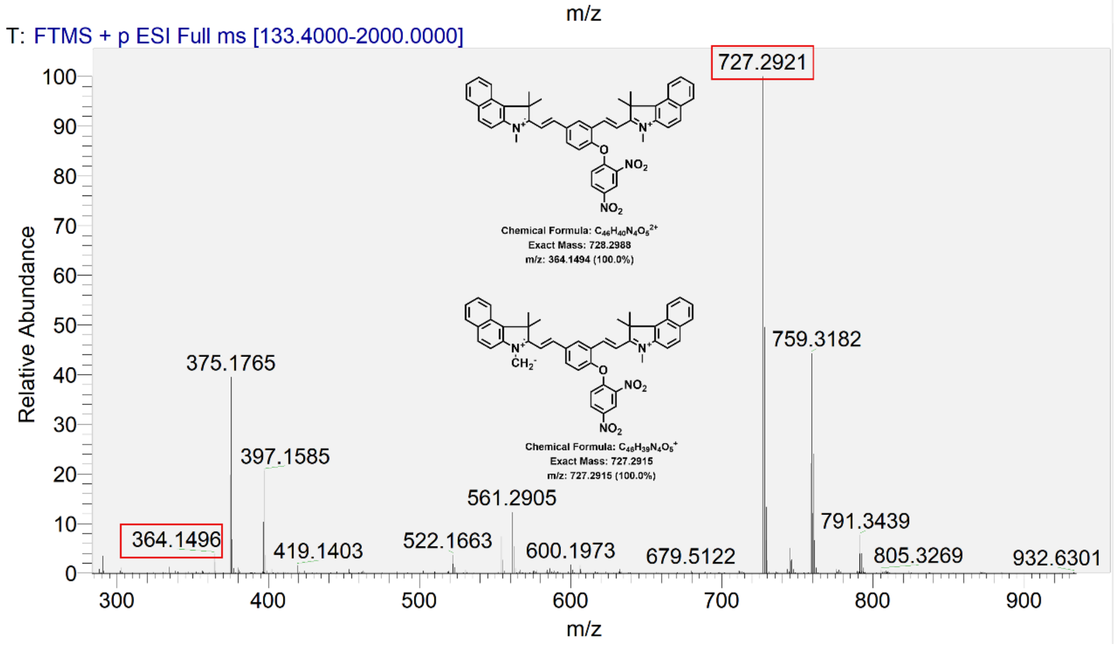

Figure S3 HRMS of FS-HS-1

2. Synthesis and characterization of FS-HS-2

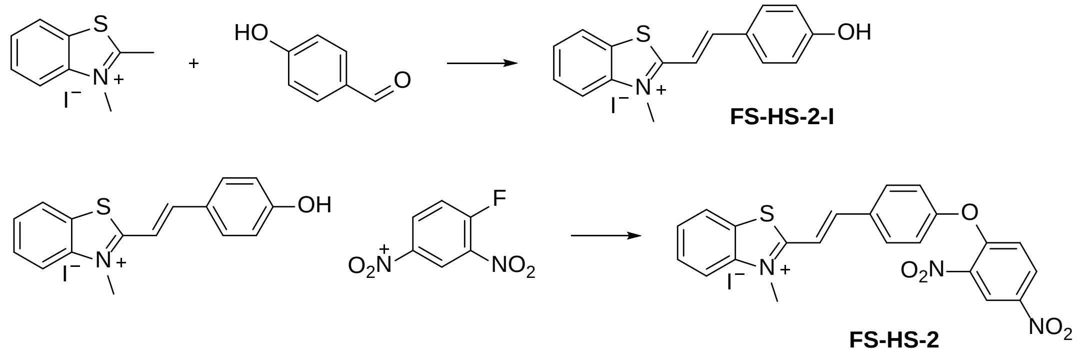

Scheme S1. Synthesis of probe FS-HS-2

8 μL of piperidine was added to 8 mL of stirred solution ethanol containing 2,3-dimethylbenzothiazol-3-ium (0.1 g, 0.34 mmol). After 10 min, p-hydroxybenzaldehyde (20 mg, 0.16 mmol) was added in 2 mL of ethanol. The reaction was refluxed at 80 °C for 3 h under nitrogen atmosphere. The reaction was cooled to room temperature and filtered to obtain 30 mg of orange powder in 68.68% yield. It was pure enough to continue the reaction.

FS-HS-2-I (1 mmol, 0.268 g), 2,4 dinitrofluorobenzene (1.2 mmol, 0.223 g), potassium carbonate (1.2 mmol, 0.165 g) were weighed in a round-bottomed flask, and anhydrous acetonitrile was added to 8 mL, and the reaction was carried out at room temperature for 8 hours. The filtrate was filtered and separated on a silica gel column to obtain 0.186 g of yellow powder with 43.2% yield.

^{1}H NMR (600 MHz, DMSO-d_6) δ 8.67 (s, 2H), 8.33 (s, 2H), 7.79 (s, 1H), 7.78 (s, 2H), 7.76 (s, 1H), 7.45 (s, 2H), 7.41 (d, J = 15.4 Hz, 2H), 7.19 (s, 2H), 6.56 (s, 2H), 3.59 (s, 6H). Chemical Formula: $C_{29}H_{24}N_3O_5^+$, MS(EI) m/z 494.17; found 494.18.

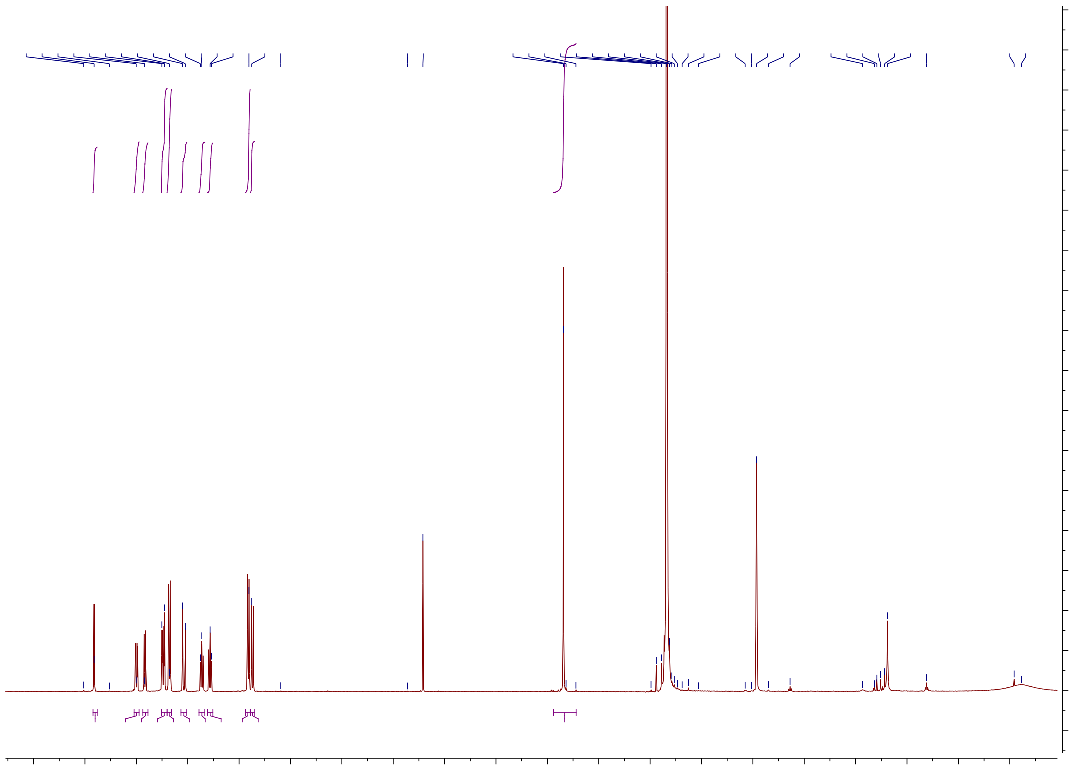

Figure S4 ¹HNMR of FS-HS-2

3. Synthesis and characterization of FS-HS-1-P

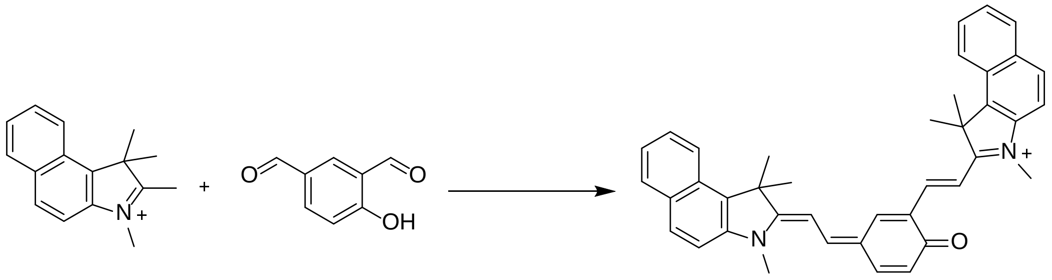

Scheme S2. Synthesis of probe FS-HS-1-P

Complex 1a (0.209 g, 1 mmol) and 4-hydroxyisophthalaldehyde (300 mg, 2 mmol) were placed in a mixed solution of n-butanol/benzene (v/v, 7/3), refluxed in an argon environment for 3 hours, the precipitate was collected, and the eluent of ethylene chloromethane:methanol = 1:15 was separated by silica gel column, and the product was obtained by 242 mg with a yield of 43.24%.

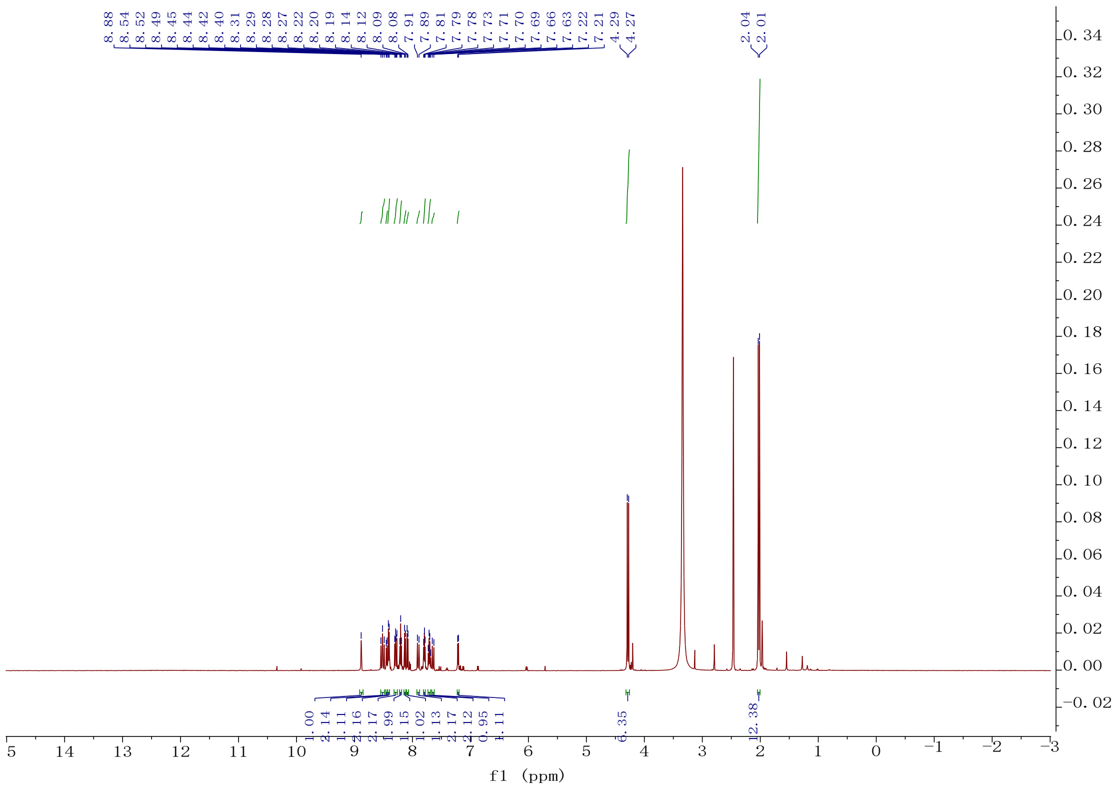

Figure S5 ^{1}HNMR of FS-HS-1-P

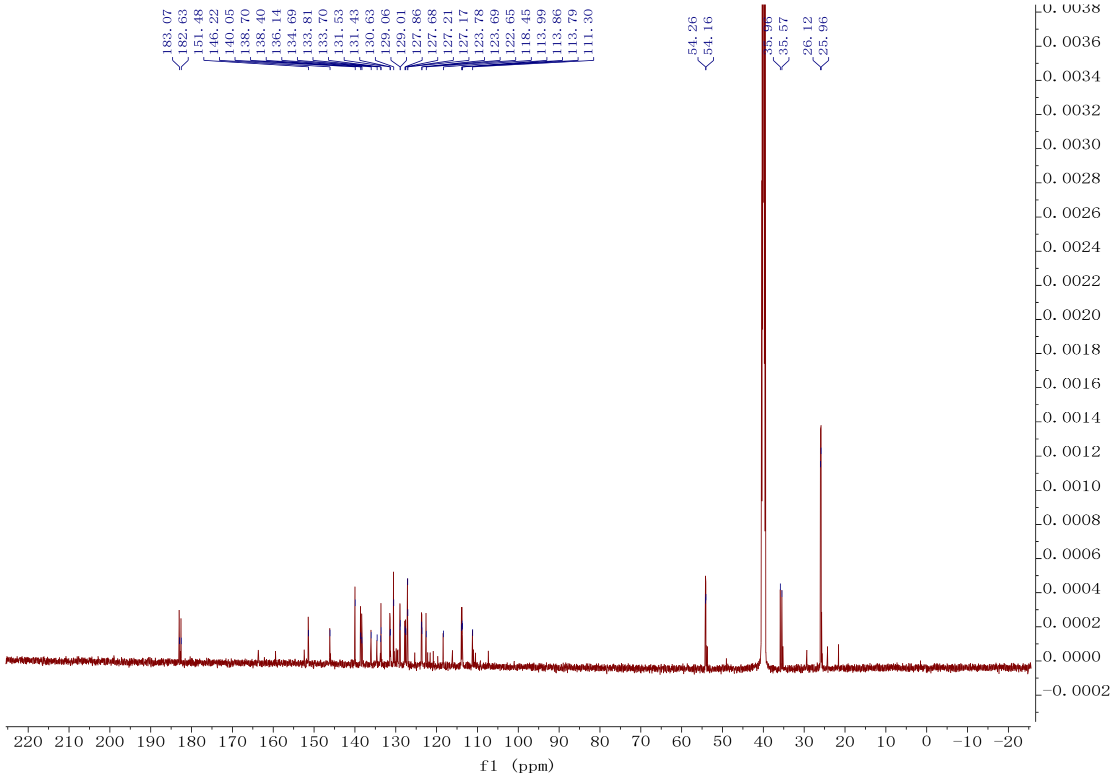

Figure S6 ^{13}CNMR of FS-HS-1-P

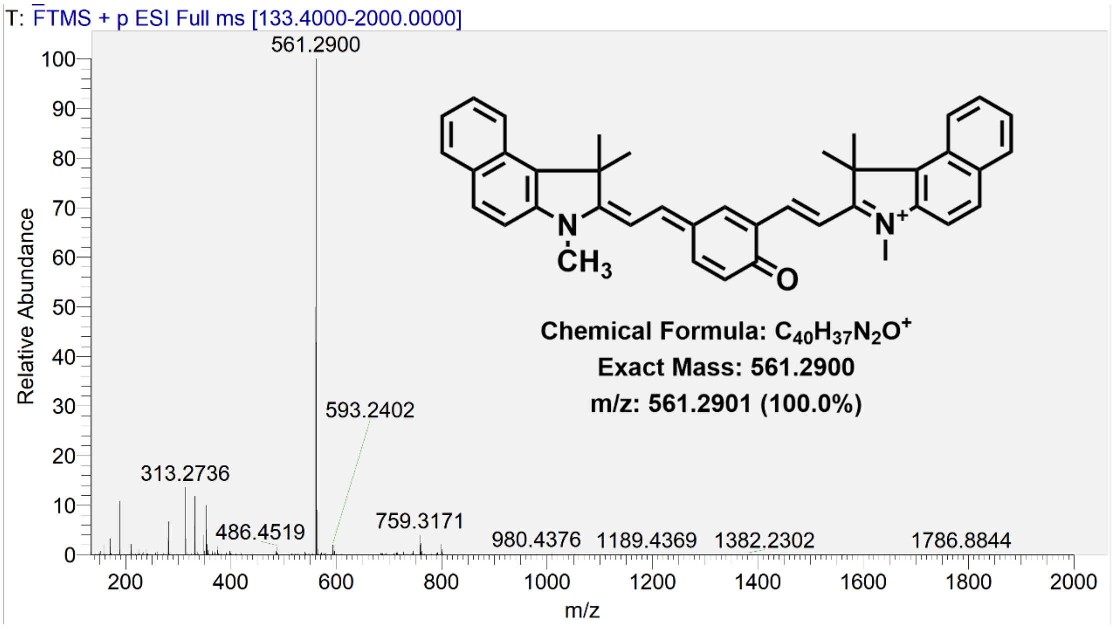

Figure S7 HRMS of FS-HS-1-P

4. Spectroscopic experimental results of FS-HS-2

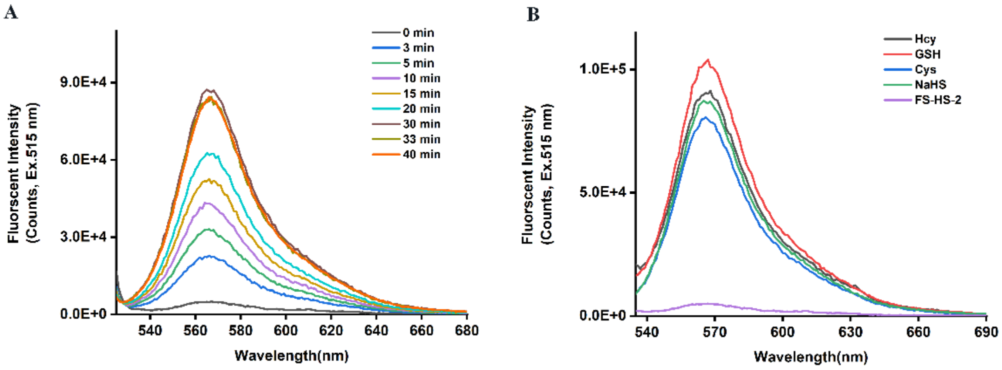

Figure S8. Behaviors of FS-HS-2 (10 μM) towards NaHS (100 μM): The response times (A); Fluorescence images of FS-HS-2 on biothiols (100 μM) respectively (B).

The selectivity and response times of FS-HS-2 were performed at this setting: λex=515 nm / λem=566 nm, Step: 1.00 nm, Dwell: 0.1s，Repeats: 1, Bdw= 4 nm.

5. Colorimetric potential of FS-HS-1

[NaHS](μM) 0 5 10 15 25 50 100

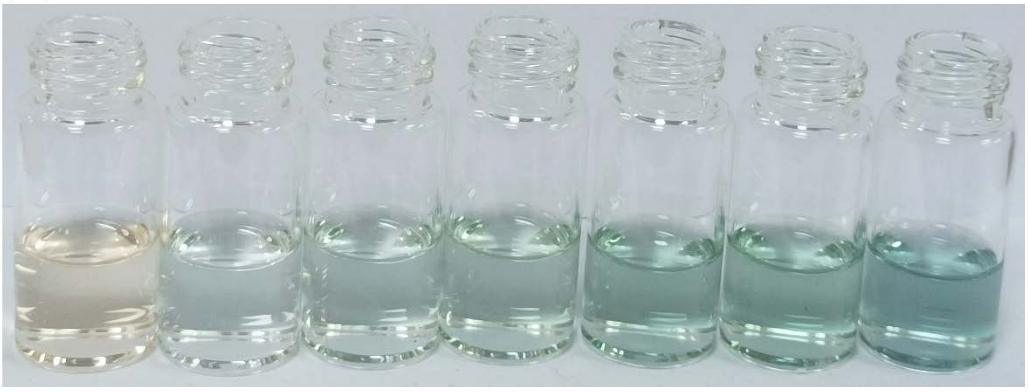

Figure S9. Colorimetric behaviors of FS-HS-1 (10 μM) towards NaHS (0, 5,10, 15,25, 50, 100 μM).

6. Comparison with the reported H_2S-specific fluorescent probes

Table S1 Comparison of FS-HS-1 with the reported H_2S-specific fluorescent probes

Compound	Compound's structure	Solvent	λ_{ex} (nm)	λ_{em} (nm)	Response time (min)	Detection limit (μM)	Application
FS-HS-1	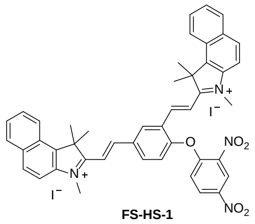	PBS/CH_3CN (V/V, 6/4)	605	715	< 2	$4.47x10^{-3}$	L929 cell
Rho-HS [39]	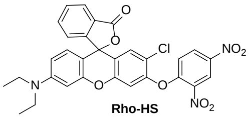	PBS/DMSO (V/V, 9/1)	510	561	15	$5x10^{-1}$	MCF-7 cells Caenorhabditis elegans
Fur-SH [40]	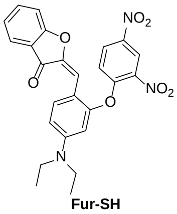	PBS/DMSO (V/V, 9/1)	510	570	5	$5x10^{-2}$	MCF-7 cells Zebrafish
TPABF-HS [41]	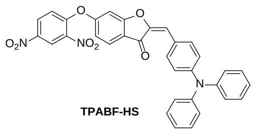	PBS	445	540	100	$4.2x10^{-1}$	MCF-7 cells

Compound	Compound's structure	Solvent	λ_{ex} (nm)	λ_{em} (nm)	Response time (min)	Detection limit (μM)	Application
NRDNP [42]	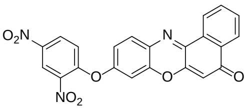 NRDNP	PBS/DMSO (V/V, 9/1)	582	612	10	1.9x10^{-2}	RAW264.7 cells
CMHS [43]	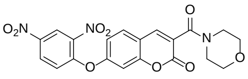 CMHS	PBS/CH_3CN (V/V, 9/1)	406	455	30	2.31x10^{-1}	HeLa cells
XZH1-H_2S [44]	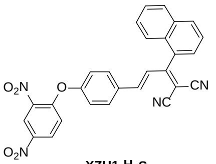 XZH1-H_2S	PBS/DMSO (V/V, 8/2)	-	585	30	1.8x10^{-1}	MCF-7 cells Mice
QVPB-DNP [45]	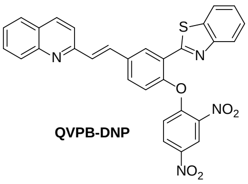 QVPB-DNP	PBS/DMSO (V/V, 3/7)	445	605	3	1.12x10^{-1}	Pork Eggs
Hcy-HSP [46]	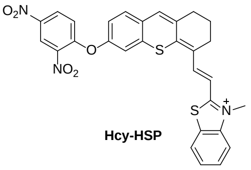 Hcy-HSP	PBS/DMSO (V/V, 19/1)	720	787	20	9x10^{-2}	SH-SY5Y cells
